# Endotracheal suture through extending tracheostoma for post-tracheostomy tracheal laceration: a case report

**DOI:** 10.1186/s13256-023-03845-w

**Published:** 2023-05-10

**Authors:** Changsung Han, Eunji Kim, Jonggeun Lee, Hyo Yeong Ahn

**Affiliations:** Department of Thoracic and Cardiovascular Surgery and Biomedical Research Institute, Pusan National University School of Medicine, Pusan National University Hospital, 305, Gudeok-Ro, Seo-Gu, Busan, 602-739 Republic of Korea

**Keywords:** Tracheal injury, Tracheostomy, Endotracheal suture

## Abstract

**Background:**

Tracheal laceration is very rare but can be life-threatening if proper treatment is not provided. The general concept for the management of tracheal laceration is surgical repair through cervical incision or via thoracotomy. However, in the case of tracheal laceration after tracheostomy, tracheostoma could be extended to avoid urgent surgical repair and additional incision.

**Case presentation:**

A 30-year-old Asian woman suffered intracerebral hemorrhage. Tracheostomy was necessary for prolonged ventilator care. While tracheostomy was performed, the posterior tracheal wall was torn. After observing that, we reinserted endotracheal tube through the oral orifice. Following bronchoscopy showed torn posterior tracheal wall. The tearing wound was 5–6 cm in length, from the middle to distal parts of the trachea. We used minimally invasive procedure for extending the already existing tracheostoma.

**Conclusions:**

In the case of tracheal laceration related to tracheostomy, a new incision is not necessary because the tracheal opening already exists. Using the extended tracheostomy technique, tracheal laceration can be repaired by endotracheal suture method.

## Background

Tracheal laceration is a rare complication that can occur during intubation or tracheostomy [1]. Most lacerations occur longitudinally, at a membranous portion (a connection between cartilage rings) of the trachea [1, 5]. The incidence of tracheal laceration is approximately 1 per 2000 intubations; however, the exact statistical probability of all tracheal lacerations cannot be defined [1, 2, 4]. If tracheal laceration is suspected, emergency bronchoscopy should be performed for diagnosis [2]. Treatment is tailored according to the location and size of the torn tracheal wall [2, 3]. Unless early correction through surgical repair is performed, tracheal laceration can progress to various catastrophic situations [4]. Herein, we report a case of post-tracheostomy tracheal laceration that was successfully managed using extended tracheostomy.

## Case report

We report the case of a 30-year-old Asian woman, with no past medical history, diagnosed with locked-in syndrome due to multiple postpartum infarctions. Intracerebral hemorrhage and intraventricular hemorrhage were confirmed during follow-up, after which she was admitted to the intensive care unit. Long-term mechanical ventilation care was expected after intubation; the patient was thus referred to the thoracic surgery department for tracheostomy.

**Fig. 1:**
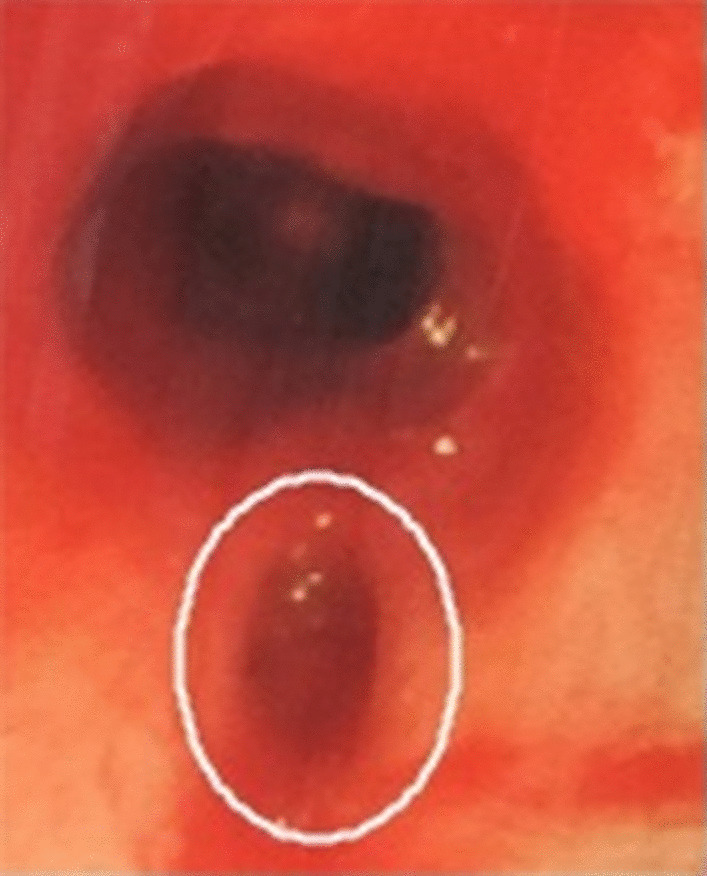
Emergent bronchoscopy, showing the torn posterior tracheal wall (circle)

Tracheostomy was performed in the usual manner (Fig. 1). In this case, complications such as subcutaneous emphysema or pneumo-mediastinum did not occur; however, tidal volume did not shift to the lung once the tube was connected to a mechanical ventilator. After noting that the ventilator was not working appropriately, the endotracheal tube was reinserted through the oral orifice. Then, through bronchoscopy, we found that the posterior tracheal wall was torn. The tearing wound was 5–6 cm in length, from the middle to distal parts of the trachea (approximately, third to fifth tracheal rings). At this point, following general guidelines, we should have considered right thoracotomy for management. However, we performed extending tracheostoma technique and endotracheal suture.

**Fig. 2:**
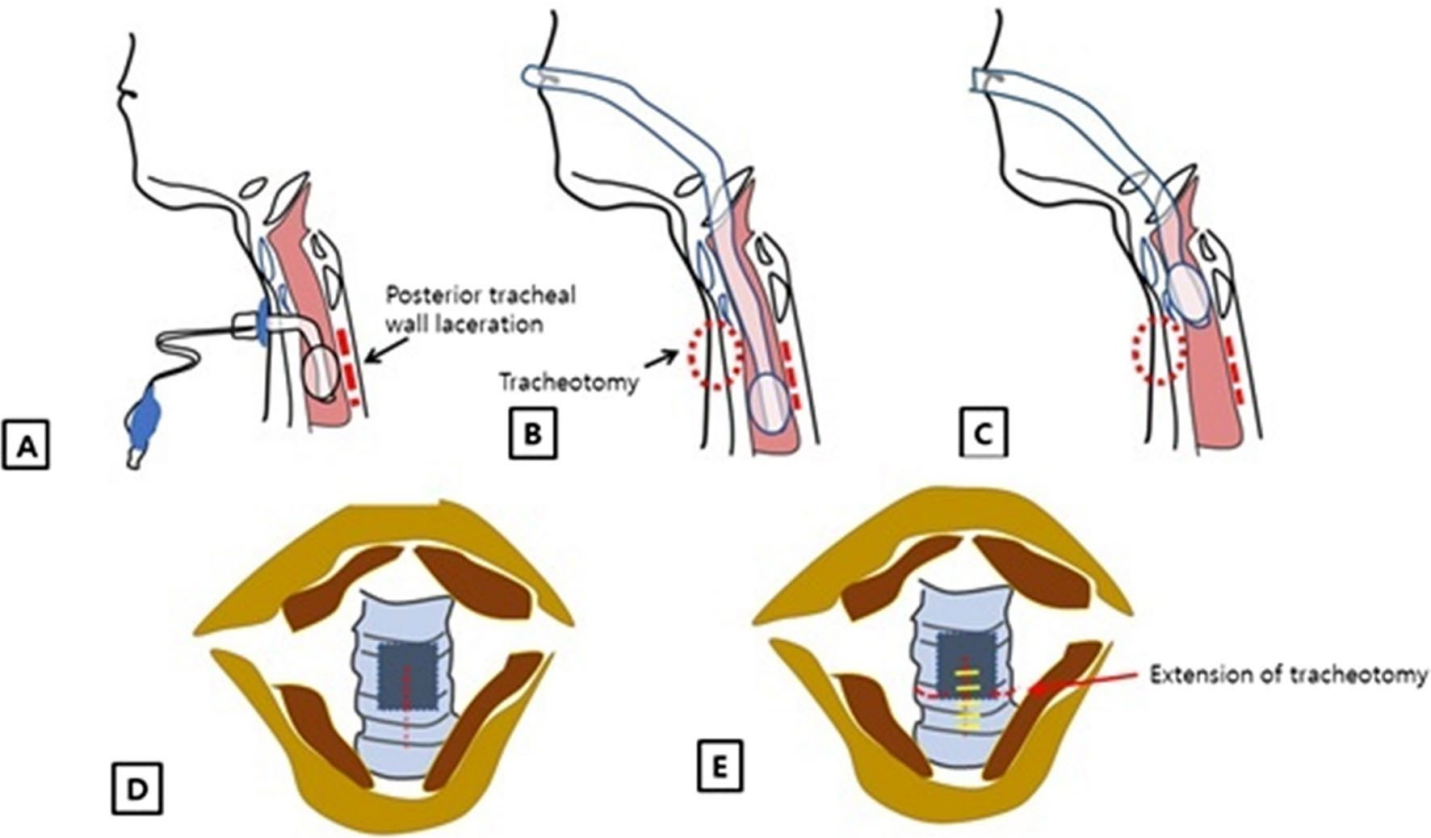
Extended tracheostomy with trans-tracheal suture. **A** The torn posterior tracheal wall during tracheostomy tube insertion. **B** The reinserted ETT tube and torn site, confirmed by bronchoscopy. **C** The torn site, exposed by pulling the ETT tube up. **D** The torn site in the posterior tracheal wall (indicated by the red line). **E** The extended tracheostoma (indicated by the horizontal red line). The torn site under direct vision (indicated by the yellow line)

After examining the torn tracheal wall via bronchoscopy (Fig. 2), the endotracheal (ETT) tube was lifted from tracheostoma level, and the tracheostoma was extended on both sides to expose the laceration area. After the area was confirmed, under direct vision, suturing was started from the superior part, with vicryl 4–0, using an open needle holder. While lifting the thread, the trachea under the opening level was exposed as much as possible, and continuous suture was performed downward. After suturing was complete, the tracheostomy tube was placed slightly below the lesion, facilitating mechanical ventilation care.

**Fig. 3:**
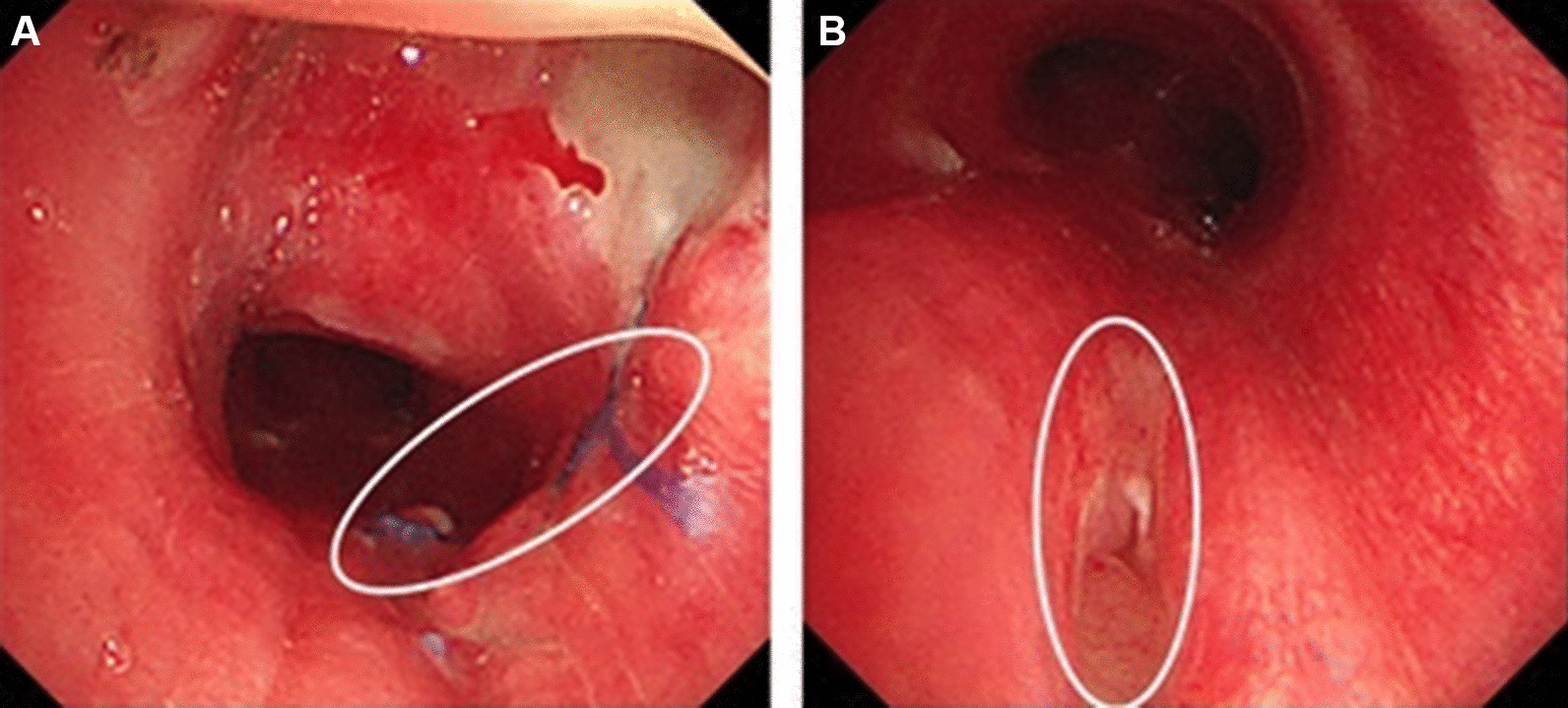
**A** The healed tracheal lesion (circle), **B** 7 days after suture with 4-0 vicryl (circle)

After 7 days, antibiotics for pneumonia were discontinued. There were no postoperative complications (Fig. 3).

On day 13, the patient was moved to the general ward, and her first tracheostomy tube change was performed 2 weeks later. No oxygen requirements were present. She was then transferred to a rehabilitation hospital.

## Discussion

Tracheal laceration is a rare complication that occurs during the process of intubation or tracheostomy. It can have fatal consequences [1, 2]. Tracheal laceration can be diagnosed via bronchoscopy, which can confirm the location and size of the tear wound in the tracheal wall [1–4]. Even if the lesion cannot be visually confirmed by bronchoscopy, a lesion should be suspected if subcutaneous emphysema, pneumothorax, or pneumo-mediastinum suddenly occurs [1–4]. If mechanical ventilation is ineffective, despite a well-placed tracheostomy tube (as in our case), examinations must be performed to identify whether the tracheal wall has been torn.

If tracheal laceration management is not performed in a timely manner, laceration may progress to descending mediastinitis and worsen the patient’s clinical condition [1, 4]. Treatment of tracheal lacerations is divided into conservative versus surgical treatments, depending on the size and location of the laceration involved [1, 3, 4]. Conservative treatment can be used if the longitudinal length of the laceration is shorter than 2 cm and the patient’s vital signs are stable. Timely surgical treatment should be performed if the longitudinal length of the laceration is longer than 2 cm and the patient’s vital signs are unstable [2, 4].

Since the surgical approach is dependent on the size and location of the tear wound, if the upper and middle parts of the trachea are affected, left cervical incision or trans-tracheal repair is chosen; if the distal trachea or main bronchus are affected, right thoracotomy is generally chosen [1, 3, 4].

In this report, making a new incision was not necessary due to an already existing tracheal opening. The tracheostoma was extended bilaterally, exposing the lacerated tracheal wall, and endotracheal suture was performed. During the suturing process, clashes between instruments could occur and it might be difficult to expose the lesion. However, by extending the previous incision, the tearing point can be repaired under direct vision. In addition, tagging of the first suture site helped us maintain tension, secure the previous suture, and easily suture the lesion continuously with a “no touch” technique.

## Conclusion

The minimally invasive method performed in this case might be a useful treatment for patients who are expected to have long-term mechanical ventilation care. Endotracheal suture through tracheostoma could reduce the incidence of additional morbidity or mortality caused by general anesthesia, broadening treatment options in cases of post-tracheostomy tracheal laceration.

## Data Availability

The data used and analyzed in this report are located at Pusan National University Hospital, Pusan, Korea and the data are available from the corresponding author on reasonable request.
